# Nonsuppressible Oral Dexamethasone Suppression Tests but Not Cushing Syndrome

**DOI:** 10.1155/2016/3684287

**Published:** 2016-03-22

**Authors:** Abilash Nair, Atul Dhingra, Anjana Gopi, Viveka P. Jyotsna

**Affiliations:** ^1^Department of Endocrinology and Metabolism, All India Institute of Medical Sciences, New Delhi 110029, India; ^2^Department of Pediatrics, ESIC Model & Super Specialty Hospital, Asramam, Kollam, Kerala 691002, India

## Abstract

In spite of the presence of definitive diagnostic criteria to diagnose Cushing syndrome diagnosis may become challenging. We report a young female with mild clinical features of Cushing syndrome, who had nonsuppressible oral dexamethasone suppression tests; also she had a suspicious pituitary lesion. She underwent pituitary surgery and a pituitary microadenoma (non-ACTH staining) was removed. Now she had come to us with similar complaints to those before. Again she had nonsuppressible oral dexamethasone suppression tests. As the diurnal variation of serum and salivary cortisol was maintained and urinary free cortisol was normal, further evaluation with IV dexamethasone suppression test was performed which clearly ruled out Cushing syndrome. The patient was not on any medicines known to alter dexamethasone metabolism. Fat malabsorption was also ruled out using appropriate tests. The reason for this discrepancy is thought to be altered (increased) metabolism of dexamethasone in this patient as it is widely variable in the general population.

## 1. Introduction

There are definite criteria for diagnosis of Cushing syndrome. Still, in patients with suggestive symptoms and borderline elevations of serum cortisol the diagnosis becomes challenging. In such instances specialized investigations to avoid unnecessary surgery and morbidity may be required.

## 2. Case History

We report a case of a twenty-five-year-old female who initially presented to an endocrinology center at the age of 16 yrs with history of weight gain of 10 kg over 3 years, mild proximal muscle weakness, irregular menstrual cycles (polymenorrhoea), and intermittent right sided headache. The patient was not on any herbal or naturopathic treatment and was not taking oral contraceptives or any other medicines. The investigations showed unsuppressed overnight and low dose 48 hr dexamethasone suppression test (Table 1) and 24 h urinary free cortisol was not measured. Also MRI sella showed a right sided pituitary microadenoma of size 5 mm × 5 mm. She underwent transsphenoidal removal of the suspected adenoma at 17 yrs of age with postoperative CSF rhinorrhea for four months. The histopathology showed a pituitary adenoma but no ACTH staining areas. She did not experience a gross difference in her symptoms after the surgery.

After the surgery the patient was started on steroid replacement and referred to our clinic for monitoring of therapy. Prednisolone was used for supplementation in a dose ranging from 10 to 2.5 mg/day. She had hypocortisolemic symptoms on tapering steroid supplementation and required supplementation for 7 years although no stimulation test is available to confirm hypothalamic-pituitary-adrenal axis hypofunction. Thyroid and gonadal axis were preserved. The irregularity of menstrual cycles continued. Steroid supplementation was carefully withdrawn 2 years prior to current investigations. Six months back she complained of recurrence of headache. She had also gained 4-5 kgs of weight over 3-4 months. There were no other symptoms suggestive of recurrence of Cushing syndrome. Physical examination showed no evidence of proximal myopathy, Cushingoid striae, or other signs favoring Cushing syndrome. The basal (morning) cortisol was elevated, overnight (1 mg) dexamethasone suppression test and low dose 48 hr (2 mg/day) dexamethasone suppression test were both nonsuppressed, and the plasma ACTH level was also high [Table 2]. As she had a previous diagnosis of “Cushing disease,” a recurrence was suspected and a dynamic contrast enhanced MRI of the sella was done which revealed a right sided delayed enhancing lesion on T1 weighted sequences suggestive of a pituitary microadenoma. The rest of the pituitary was normal on the MRI with a convex upper margin and the pituitary stalk central in position. She was admitted for a repeat transsphenoidal surgery for microadenoma, neurosurgical reference was taken, and it was planned for her to undergo surgery. On admission, as a routine, morning and midnight serum cortisol and midnight salivary cortisol tests and basal ACTH were evaluated which were normal and showed a preserved diurnal rhythm [Table 2]. This made our diagnosis suspicious. The surgery was put on hold, pending confirmation of the diagnosis.

Suspecting noncompliance to dexamethasone during testing, the low dose (2 mg/day) DST was repeated under supervision in the ward, which was again unsuppressed. A high dose (8 mg/day) DST was also done which showed >50% reduction in the cortisol from baseline, but still the cortisol was above 5 mcg/dL. A 24-hour urine free cortisol estimation was done which was normal [Table 2].

**Figure 1:**
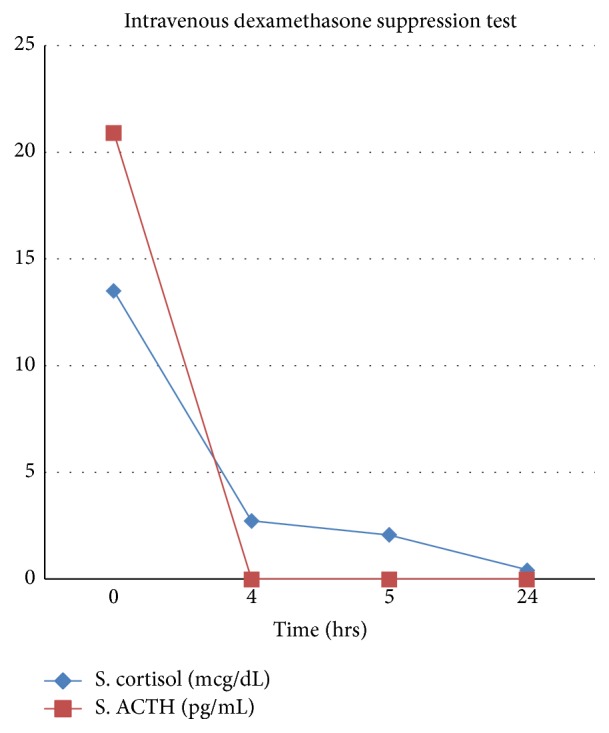
ACTH and cortisol dynamics during the intravenous dexamethasone suppression test.

Intravenous dexamethasone suppression test was done to rule out hypercortisolism. The 4 mg-over-4-hour-infusion (1 mg/hr) protocol originally suggested by Abou-Samra et al. was used for the test [1]. Samples were drawn at 0 hr, 4 hr, 5 hr, and 24 hrs of the starting of IV dexamethasone infusion. The expected response in a healthy person is 24 hr (day 2) cortisol value of <2.7 mcg/dL and in patients of Cushing syndrome is more than >4.7 mcg/L (or >20% of the baseline [0 hr]) [2]. The dynamics of cortisol during the test are shown in Table 3 and Figure 1 which was normal. Hence, Cushing syndrome was ruled out. Now, an attempt to explain the false positive dexamethasone suppression test was made. Serum levels of dexamethasone could not be measured due to lack of resources. The patient was not on treatment with antiepileptic drugs, oral contraceptives, or any other medicines known to increase metabolism of dexamethasone. The patient did not have any clinical features of malabsorption and a 24 Hr fecal fat excretion after fat loading of 75 grams/day was 0.51 g/day (normal: < 6 grams/day). The patient was discharged with symptomatic measures for headache and advice on lifestyle modification for preventing weight gain.

## 3. Discussion

Although some authors have shown midnight serum cortisol to be highly sensitive and specific for the diagnosis of Cushing syndrome [3], the current clinical practice guidelines of the Endocrine Society suggest one of the four tests with high sensitivity (24 hr urine free cortisol, midnight salivary cortisol, overnight (1 mg) DST, or low dose [2 mg/d] DST) should be used for the screening for Cushing syndrome [4]. If one of the above is positive, another of the remaining three should be done and if 2 of the four tests are positive this confirms the diagnosis of Cushing syndrome. Based on the guidelines Cushing syndrome was confirmed in the patient in the case report (unsuppressed overnight and low dose DST). MRI had also showed a lesion suggestive of microadenoma for which she underwent surgery previously and remained hypocortisolemic postoperatively probably due to surgical damage to the pituitary taking a long time to recover to normal. Additional tests in the wake of a normal diurnal rhythm of cortisol secretion made the diagnosis of Cushing syndrome unlikely and 4 mg IV DST ruled it out decisively. Thus the oral dexamethasone suppression tests were false positive and the unnecessary surgery was averted. Presentations similar to the current case can be seen in patients with cyclical Cushing's syndrome and cortisol binding globulin (CBG) excess state. In this case, as oral DSTs and IV DST which were done at a gap of just 7 days showed a marked difference in supressibility cyclical Cushing was considered unlikely. Furthermore, as IVDST showed clear-cut suppression of cortisol within hours of dexamethasone administration, CBG excess state was ruled out. IV DST is used in clinical situations where either decreased absorption of dexamethasone or its increased metabolism is suspected. It is reported that the plasma dexamethasone levels may vary widely in normal persons after oral administration for DSTs [5]. The rate of inactivation of dexamethasone by the human liver microsomes is highly variable as measured by the production of 6*β*-hydroxydexamethasone and other metabolites [6]. Simultaneous measurement of plasma dexamethasone levels and cortisol levels has been suggested to assess the adequacy of absorption of dexamethasone during DSTs, but this facility is not readily available outside the United States.

## 4. Conclusion

Through this report we point out why the diagnosis of Cushing syndrome must not be based on two oral dexamethasone suppression tests alone. A role of IV DSTs in cases where oral DSTs are contradictory to the other measures of hypercortisolism is emphasized by this case report. Before operating a case of Cushing syndrome with borderline features of hypercortisolism additional tests may be used for confirming or ruling out the diagnosis and the patient should be kept on follow-up till a definite diagnosis is reached.

## Figures and Tables

**Table 1:** Investigation summary prior to the first surgery.

Date	20 and 21/6/2006	11/8/2006	17/1/2007	16/7/2007	28/11/2007
S. cortisol, 8 am (mcg/dL)	18.12	15.76	Patient underwent TSS and removal of suspected pituitary adenoma		
S. cortisol, 4 pm (mcg/dL)	20.29				
Overnight DST (1 mg) (mcg/dL)	12.68			0.54	1.81
Low dose 48 hr DST (2 mg/day) (mcg/dL)		4.67			
High dose 48 hr DST (8 mg/day) (mcg/dL)		1.19			

**Table 2:** Investigation summary-current evaluation.

Date	Jan 2015	26/3/15	27/3/15	30/3/15 & 4/4/15 (supervised)	4/4/15	10/4/15
S. cortisol (8 am) mcg/dL	22.38	Admitted	9.63		16.09	
S. cortisol (midnight) mcg/dL			4.95		6.68	
Overnight DST (1 mg) mcg/dL	**13.20 **					
Low dose 48 hr DST (2 mg/day) mcg/dL	**15.47**			**11.96**		
High dose 48 hr DST (8 mg/day) mcg/dL				**6.39**		
Salivary cortisol midnight (normal [kit insert] < 0.42 mcg/dL)			0.306		0.217	
ACTH pg/mL			7.29		29.85	
24 Hr urine free cortisol mcg/day (normal 28.5–213.7)						110.72

**Table 3:** ACTH and cortisol dynamics during the intravenous dexamethasone suppression test.

4 mg IV DST	S. cortisol	ACTH
0 hr	13.51 mcg/dL	20.92 pg/mL
4 hr	2.72 mcg/dL	<1 pg/mL
5 hr	2.05 mcg/dL	<1 pg/mL
24 hr	0.43 mcg/dL	<1 pg/mL

## Additional Points

Diagnosis of Cushing disease must not be based on oral dexamethasone suppression tests (DSTs) alone. Assessment of diurnal rhythm, salivary cortisol, and urine free cortisol levels can help in diagnosis. There is a definite role for intravenous DST in cases where oral DSTs are contradictory to other measures of hypercortisolism.
